# ‘Emergency exit' of bone-marrow-resident CD34^+^DNAM-1^bright^CXCR4^+^-committed lymphoid precursors during chronic infection and inflammation

**DOI:** 10.1038/ncomms9109

**Published:** 2015-10-05

**Authors:** Federica Bozzano, Francesco Marras, Maria Libera Ascierto, Claudia Cantoni, Giovanni Cenderello, Chiara Dentone, Antonio Di Biagio, Giancarlo Orofino, Eugenio Mantia, Silvia Boni, Pasqualina De Leo, Antonino Picciotto, Fulvio Braido, Francesca Antonini, Ena Wang, Francesco Marincola, Lorenzo Moretta, Andrea De Maria

**Affiliations:** 1 Department of Experimental Medicine, University of Genova, Via Pastore 1, Genova 16132, Italy; 2 Center for Excellence in Biomedical Research, University of Genova, Via Pastore 1, Genova 16132, Italy; 3 Istituto Giannina Gaslini, Genova 16148, Italy; 4 Department of Transfusion Medicine, Clinical Center and Center of Human Immunology, National Institutes of Health, Bethesda, Maryland 20892, USA; 5 Department of Oncology, Johns Hopkins University, Baltimore, Maryland 21231, USA; 6 U.O.C. Malattie Infettive, Ospedale Galliera, Mura delle Cappuccine 14, Genova 16128, Italy; 7 U.O.C. Malattie Infettive, Ospedale Sanremo, Via Privata Barabino 15, Sanremo 18038, Italy; 8 Clinica Malattie Infettive, IRCCS AOU San Martino-IST Genova, Istituto Nazionale per la Ricerca sul Cancro, Largo Rosanna Benzi 10, Genova 16132, Italy; 9 SOC Malattie Infettive ASO S.S. Antonio e Biagio e C. Arrigo Alessandria, 15100, Italy; 10 U.O.C. Malattie Infettive, Ospedale Amedeo di Savoia, Torino 10149, Italy; 11 U.O.C. Malattie Infettive, Ospedale Sant'Andrea, La Spezia 19121, Italy; 12 U.O.C. Malattie Infettive, Azienda Sanitaria Locale n.2, Savona 17100, Italy; 13 Allergy and Respiratory Unit, Department of Internal Medicine, University of Genova, Via Pastore 1, Genova 16132, Italy; 14 Hepatology Unit, Department of Internal Medicine, University of Genova, Via Pastore 1, Genova 16132, Italy; 15 Sidra Medical and Research Centre, Doha P.O. BOX 26999, Qatar; 16 Department of Health Sciences, DISSAL, University of Genova, Via Pastore 1, Genova 16132, Italy

## Abstract

During chronic inflammatory disorders, a persistent natural killer (NK) cell derangement is observed. While increased cell turnover is expected, little is known about whether and how NK-cell homeostatic balance is maintained. Here, flow cytometric analysis of peripheral blood mononuclear cells in chronic inflammatory disorders, both infectious and non-infectious, reveals the presence of a CD34^+^CD226(DNAM-1)^bright^CXCR4^+^ cell population displaying transcriptional signatures typical of common lymphocyte precursors and giving rise to NK-cell progenies with high expression of activating receptors and mature function and even to α/β T lymphocytes. CD34^+^CD226^bright^CXCR4^+^ cells reside in bone marrow, hardly circulate in healthy donors and are absent in cord blood. Their proportion correlates with the degree of inflammation, reflecting lymphoid cell turnover/reconstitution during chronic inflammation. These findings provide insight on intermediate stages of NK-cell development, a view of emergency recruitment of cell precursors, and upgrade our understanding and monitoring of chronic inflammatory conditions.

## Abstract (web summary)

Lymphocytes are on high demand during chronic infections. Here, the authors show that human lymphoid precursors, normally found in the bone marrow, circulate in the blood of chronic inflammation patients and give rise to natural killer cells and other lymphocytes.

## Graphical abstract

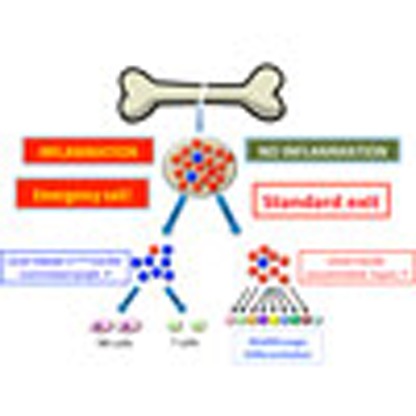

Natural killer (NK) cells originate from CD34^+^ haematopoietic stem cells (HSC) through discrete stages of development^1^,^2^. Maturation of CD34^+^ HSC into CD56^bright^CD16^+/−^ NK cells begins in bone marrow (BM) and secondary lymphoid organs^3^,^4^, is completed in the periphery where the CD56^bright^CD16^+/−^ to CD56^dim^CD16^+^ transition^5^ occurs and is followed by the acquisition of maturity molecule expression (for example, KIR, CD57,CD85j)^6^,^7^. *In vitro*, discrete maturation stages of NK cells from bone-marrow-derived CD34^+^ stem cells have been characterized^8^,^9^, while *in vivo* this aspect still eludes full understanding^3^,^10^.

In the course of acute and chronic infections including cytomegalovirus (CMV), hepatitis C Virus (HCV), HIV-1, *Mycobacterium tuberculosis* or Chikungunja virus, peripheral NK cells undergo transient or persistent modulation of triggering receptor expression, and their functional activity^4^. In HIV infection, decreased CD4^+^ T-cell numbers are paralleled by derangements of innate immunity, including altered phenotype and function of NK^11^,^12^, plasmacytoid and myeloid dendritic cells^13^. In particular, NK cells show a marked downregulation of activating receptors with consequent impaired function^14^,^15^,^16^ and an activated phenotype^17^. Notably, both NK-cell activation and altered function persist even when viremia is undetectable following successful combined antiretroviral treatment (cART)^14^,^18^,^19^,^20^ and accompany incomplete immune reconstitution^21^. Extensive alterations of NK cells occur not only during HIV infection, but also in other chronic infections including HCV^22^,^23^,^24^ and tuberculosis (TB)^25^. In addition they have been observed in latent CMV infection^26^. All these conditions differ from one another for the NK phenotype and subset distribution, but share a persistent NK-cell subset modulation/activation.

Altogether, the extent of NK-cell involvement in chronic infection/inflammation and the NK-cell origin from CD34^+^ stem cells strongly suggest the possibility of an increased NK-cell production from CD34^+^ progenitors. Indeed, an increased lymphoid cell turnover with exhaustion of CD34^+^ precursors has been shown in HIV patients with continuous viral replication^27^. Remarkably, these observations conflict with previous deuterium-labelling studies in which the NK-cell turnover appeared to be unaffected during acute Epstein-Barr Virus (EBV) and during chronic HTLV-1 infection^28^.

In an attempt to shed light on these conflicting aspects and to better understand the dynamics of NK-cell homeostasis during chronic infections, we analysed potential NK precursors circulating in peripheral blood (PB).

We found relevant proportions of a CD34^+^DNAM-1^bright^CXCR4^+^ common lymphoid precursor in patients with different chronic infections. In healthy donors (HDs), these cells were barely detectable in PB and resided in BM. Cultured CD34^+^DNAM-1^bright^CXCR4^+^-generated NK cells characterized by a mature phenotype and function. Remarkably, these precursors were also detected in PB of patients with chronic inflammatory diseases without infection (chronic obstructive pulmonary disease (COPD) and pyogenic arthritis, pyoderma gangrenosum and acne (PAPA) syndrome).

## Results

### Identification of Lin^−^CD34^+^DNAM-1^bright^ cells in HIV patients

**Figure 1:**
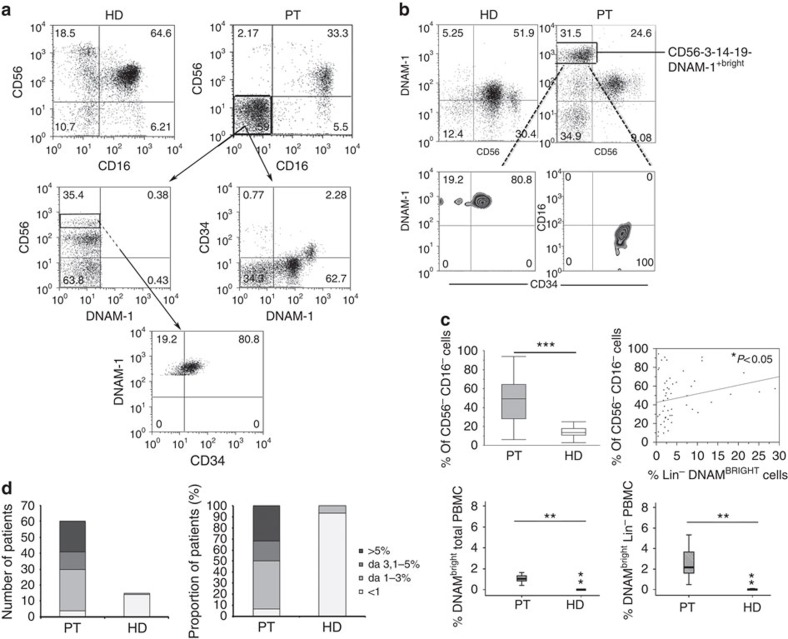
**Identification of increased proportions of Lin^−^CD226(DNAM-1)brightCD34**^+^ **cells in PBMC of patients with chronic infection.** (**a**) Flow cytometric analysis of CD56^−^CD16^−^ cells proportion in fresh PBMCs from HIV-infected patients and HD donors. In CD3^−^CD14^−^CD19^−^-gated PBMCs different proportions of CD56^−^CD16^−^ were observed in HIV-infected patients (PT) compared with healthy donors (HD). In HIV patients, CD56^−^CD16^−^-gated cells expressed DNAM-1 and CD34. Representative of 20 experiments. (**b**) Flow cytometric analysis of DNAM-1 expression on fresh PBMCs from HIV-infected patients and HD donors. DNAM-1^bright^CD56^−^ cells are observed in HIV-1 infected patient CD3^−^CD14^−^CD19^−^-gated PBMCs (upper row). DNAM-1^bright^CD56^−^ cells express CD34 but do not express CD16 (FcγRIII) (lower row). Patients (PT); healthy donors (HD). Representative of 20 experiments. (**c**) Analysis of CD56^−^CD16^−^Lin^−^ cells in fresh PBMCs from HIV-infected patients versus HD. Increased Lin^−^CD56^−^CD16^−^ cells in fresh HIV PBMC (left panel, upper row). The proportion of CD56^−^CD16^−^ cells in HIV-patient PBMCs correlates with the proportion of Lin^−^DNAM^bright^ cells. (Spearman's test *P<0.05, *r*^2^ 0.8, upper right panel). The proportion of DNAM^bright^CD56^−^ cells is increased in HIV patients in total fresh PBMCs and in CD3^−^CD14^−^CD19^−^-gated PBMCs compared with uninfected donors (lower row, left and right panel). Box-plot analyses indicate twenty-fifth and seventy-fifth percentiles with median line; vertical lines express s.d.; **P<0.01, ***P<0.0001, Mann–Whitney *U*-test. Representative of 20 experiments. (**d**) Increased proportions of DNAM^bright^CD56^−^ cells are detected in HIV patients. Left panel: increased n° of patients with given proportions of circulating DNAM-1^bright^CD56^−^ cells compared with HD (*χ*^2^-test=14.056, *P*=0.00283). Right panel: proportions of patients with high proportions of circulating DNAM^bright^CD56^−^ cells versus HD.

We first studied PB mononuclear cells (PBMC) from HIV-1 patients on cART since this condition is a paradigm of chronic low-level inflammation despite control of peripheral viremia. Analysis of CD3^−^14^−^19^−^-gated PBMC revealed relevant proportions of CD16^−^CD56^−^ cells, that is, not belonging to the T/B/monocyte/NKT/NK-cell lineages. The proportion of these CD16^−^CD56^−^ cells was much higher in HIV-infected patients than in HD (***P<0.0001, Fig. 1a,c upper left panel). A preliminary analysis of a battery of lymphoid markers revealed the presence of DNAM-1^bright^ (CD226) cells in this subset. These (Lin^−^)CD56^−^DNAM-1^bright^ cells were very low level or not detectable with standard flow cytometry in HD PBMC(Fig. 1b, upper row). The proportions of CD56^−^DNAM-1^bright^ and CD56^−^CD16^−^ cells were directly correlated (*P*=0.04, Spearman test, Fig. 1c upper right panel), thus suggesting that they may contain a shared cell population. We next tested the possibility that (Lin^−^)CD56^−^DNAM-1^bright^ PBMC could represent either circulating NK cells with an unusual CD56^−^ phenotype or Lin^−^ cells migrated from BM or other lymphoid organs. To this end, CD3^−^14^−^19^−^CD56^−^DNAM-1^bright^ cells were analysed by multiparameter flow cytometry, which revealed the expression of CD34 and lack of CD16 antigens (Fig. 1b and Supplementary Fig. 1A). These data indicate that Lin^−^CD56^−^DNAM-1^bright^ cells are indeed Lin^−^CD34^+^DNAM-1^bright^ cells.

Using a reverse flow cytometric strategy with Lin^−^ selection stain (which includes anti-CD3, -CD19, -CD20, -CD14, -CD16, -CD56 monoclonal antibodies (mAbs); Supplementary Fig. 1B) the presence of DNAM-1^bright^ cells among CD34^+^ PBMC in these patients was further confirmed. Indeed, 78% (range 41–91%) of Lin^−^CD34^+^ cells were DNAM-1^+^. Thus, Lin^−^56^−^DNAM-1^bright^ cells represent CD34^+^DNAM-1^bright^ precursors.

To provide an estimate of the frequency and size of this cell subset, we next evaluated their proportions in samples obtained from a group of HIV-infected, successfully cART-treated, patients and compared them with that of HDs. In HDs, Lin^−^DNAM-1^bright^CD34^+^ cells represented <1% of PBMC in 13 out of 15, and 1–3% in 2 of 15 donors (Fig. 1d). Remarkably, in view of the limits of standard flow cytometry on unfractionated samples, we could not exclude very low levels of these cells in the circulation (for example, <0.5–1%) in all HD, This more precise lower-limit quantification would require the routine application of cell concentration or limiting dilution methods. In contrast, Lin^−^DNAM-1^bright^CD34^+^ cells were present in a much higher proportion of HIV patients (Fig. 1d, left panel, *χ*^2^-test=14.056, *P*=0.00283). In fact, >30% of patients had >5% of DNAM-1^bright^CD34^+^ cells, in some cases reaching as much as 30–40% of Lin^−^gated cells (Fig. 1d, right panel). Accordingly, the median proportion of peripheral Lin^−^DNAM-1^bright^CD34^+^ cells was considerably higher in HIV-infected patients (**P=0.001, Mann–Whitney; Fig. 1c, bottom row).

### Phenotypic and transcriptional characteristics

**Figure 2:**
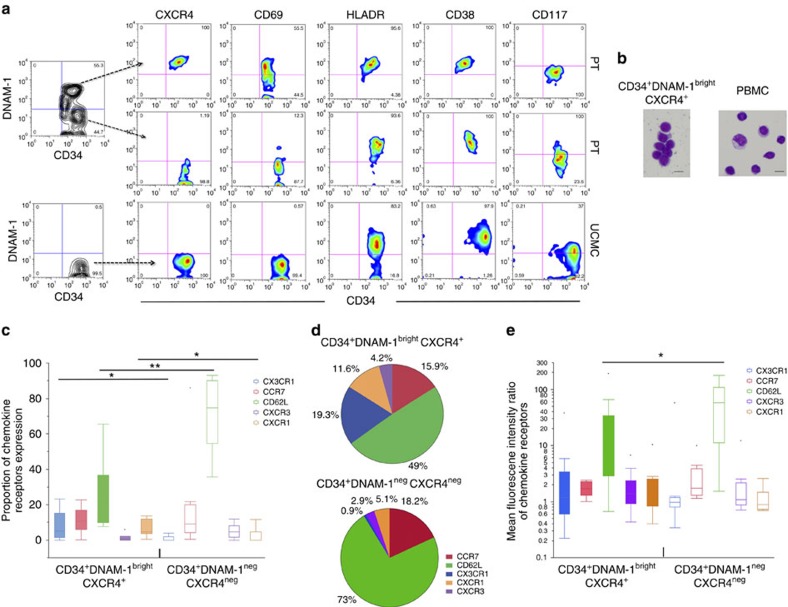
**Characterization of Lin**^**−**^**CD34**^**+**^**DNAM-1**^**bright**^ **PBMC by flow cytometry.** (**a**) Differences in receptor expression in Lin^−^DNAM-1^bright^CD34^+^ PBMC versus CD34^+^ cord blood cells. Flow cytometric dot-plot analysis of patient (PT) PBMC (upper and middle rows) and UCMC (lower row). Flow cytometric surface molecule analysis is reported for Lin^−^DNAM-1^bright^CD34^+^ PBMC (upper row), CD34^+^DNAM-1^−^ PBMC of HIV patients (middle row) and CD34^+^DNAM^−^ UCMC (lower row). Left contour dot plots show gating selection to analyse both CD34^+^ PBMC subsets. Representative of 20 experiments. (**b**) Morphology of Lin^−^DNAM-1^bright^CD34^+^ PBMC. Magnetic bead-enriched CD34^+^ PBMC were sorted by flow cytometry into CD34^+^DNAM-1^bright^CXCR4^+^ cells centrifuged on a glass slide fixed, and stained by May–Grunwald Giemsa stain. Images were acquired by Olympus BX51 Microscope & DP70 Digital Camera System. Scale bar, 7.5 μm. (**c**) Chemokine receptor expression by Lin^−^DNAM-1^bright^CD34^+^ PBMC. Flow cytometric analysis of PBMC. Lin^−^ cells were gated for DNAM-1^bright^ and CD34 expression and chemokine receptor expression was analysed on CD34^+^DNAM-1^bright^ and on CD34^+^DNAM-1^−^cells. Box-and-whiskers representation of data shows increased proportions of CX3CR1^+^ and CXCR1^+^ and lower CD62L expression on CD34^+^DNAM-1^bright^ cells. *P<0.05; **P<0.01 (Mann–Whitney *U*-test). (10 different experiments/patients). (**d**) Relative expression of chemokine receptors by CD34^+^CXCR4^+^DNAM-1^bright^ and CD34^+^DNAM-1^−^cells. Pie-chart representation of the relative frequency of chemokine receptor expression on CD34^+^ PBMC. Numbers express the overall proportion of positive cells relative to other chemokine receptors using the median values of expression of each chemokine receptor. (10 different experiments/patients). (**e**) Chemokine receptor density expression by Lin^−^DNAM-1^bright^CD34^+^ PBMC. Flow cytometric analysis of PBMC. Lin^−^ cells were gated for DNAM-1^bright^ and CD34 expression, and chemokine receptor expression was analysed on CD34^+^DNAM-1^bright^ and on CD34^+^DNAM-1^−^cells. Data are expressed as Mean fluorescence intensity ratios (MFIr). Box-and-whiskers representation of data shows lower CD62L molecule density expression on CD34^+^CXCR4^+^DNAM-1^bright^ cells. *P<0.05; **P<0.01 (Mann–Whitney *U*-test). (10 different experiments/patients).

To further characterize PB Lin^−^CD34^+^DNAM-1^bright^ cells, additional informative surface markers were analysed and compared with Lin^−^CD34^+^ stem cells isolated from umbilical cord blood (UCB) mononuclear cells (UCMC). Flow cytometric analysis showed that, different from CD34^+^ PBMC, CD34^+^ UCMC do not express DNAM-1. Additional differences were detected in surface molecule expression including CD69 and CXCR4 that are expressed only on CD34^+^DNAM-1^bright^ cells, and CD117 expressed only on CD34^+^UCMC (Fig. 2). On the other hand, CD34^+^DNAM-1^bright^ and UCMC-derived CD34^+^DNAM-1^−^ cells shared the expression of CD38, CD244(2B4), HLA-DR. Moreover, they both lacked expression of CD161, CD45RA, p75/AIRM1 (Siglec-7), CCR5, CCR4 and CD85j (LIR1-NFAT). (Table 1, Supplementary Fig. 2). Morphological analysis performed after cell sorting revealed their lymphocytic morphology (Fig. 2b).

Analysis of chemokine receptor expression (CX3CR1, CXCR3, CXCR1, CD62L and CCR7) on Lin^−^CD34^+^DNAM-1^bright^ cells showed differences as compared with CD34^+^DNAM-1 cells in the same patients as determined by flow cytometric analysis. Thus, while the expression of CCR7 and CXCR3 was comparably low in the two subsets, higher proportions of CX3CR1^+^ and CXCR1^+^ and lower proportions of CD62L^+^ cells were detected in Lin^−^CD34^+^DNAM-1^bright^ cells (Fig. 2c,d), with lower molecule density by mean fluorescence intensity (MFI) analysis only of CD62L (Fig. 2e). Based on this chemokine receptor expression pattern, CD34^+^DNAM-1^bright^CXCR4^+^ cells have the potential of trafficking not only into lymph nodes or gut-associated lymphoid tissues via CD62L/L interactions, but also (or rather) to peripheral inflamed tissues along fractalkine or IL-8 gradients. Overall, as shown by pie-chart representation of chemokine receptor expression, a relatively higher proportion of CD34^+^DNAM-1^bright^CXCR4^+^ cells express receptors other than CD62L as compared with CD34^+^DNAM-1^−^ cells (Fig. 2d).

No expression of CD1a, nor of cytoplasmic CD3, CD5, CD25, NKG2D or TdT could be detected on Lin^−^CD34^+^DNAM-1^bright^ cells, while a small fraction was CD10^+^ (median 4%; range 0.5–20%). With regard to alpha4/beta7 integrin expression beta7 was expressed on 24% of CD34^+^DNAM-1^bright^ cells, and 7% of the cells (29% of beta7^+^) were alpha4/beta7^+^.

**Figure 3:**
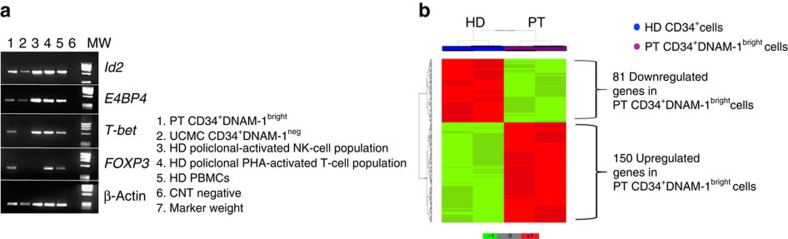
**Transcriptional analysis of CD34**^**+**^**DNAM**^**bright**^ **cells reveals different transcriptional signatures compared with cord blood CD34**^**+**^**DNAM**^**neg**^ **cells.** (**a**). Transcription factor analysis of CD34^+^DNAM-1^bright^ cells by RT–PCR. PCR products were separated by electrophoresis on a 1.5% agarose gel and visualized by ethidium bromide staining. CD34^+^DNAM-1^bright^ cells express Id2, E4BP4, T-bet and FOXP3, while UCMC CD34^+^(DNAM-1^−^) do not express T-bet and FOXP3. (**b**) Supervised cluster analysis. Supervised cluster analysis was based on the 231 genes differentially expressed (Student's *t*-test *P* value<0.01) between peripheral CD34^+^DNAM-1^bright^ cells derived from six repeated peripheral blood patient samples (PT) and from two UCMC samples from healthy donors (HD).

Since, according to their phenotype, Lin^−^CD34^+^DNAM-1^bright^ cells could represent a subset of progenitors at a stage of differentiation different from CD34^+^DNAM-1^−^ CB HSC, the two populations were purified by cell sorting (>99% purity). Their expression of transcription factors was comparatively analysed by reverse transcription–PCR (RT–PCR). PB CD34^+^DNAM-1^bright^ cells expressed Id2, E4BP4, T-bet and FOXP3, while CD34^+^(DNAM-1^−^) did not express T-bet and FOXP3 (Fig. 3a). Both CD34^+^ cells did not express GATA-1 mRNA (Supplementary Fig. 3A). Therefore, on the basis of their transcription factor expression, CD34^+^ DNAM-1^bright^ cells present in the PB of HIV patients may represent committed lymphoid precursors (CLP). Based on these data, we further performed a full transcriptional characterization of these cells. To this end, multiple purified samples from UCMC and patient PBMC were evaluated by microarray analysis. Comparative transcript expression analysis using Student's *t*-test (cut-off *P*<0.01) identified 231 genes differentially expressed by the two CD34^+^-cell populations (Fig. 3b). Of these, 150 transcripts were upregulated (Supplementary Table 1) and 81 were downregulated (Supplementary Table 2) in peripheral CD34^+^DNAM-1^bright^ cells from HIV-infected patients. Ingenuity Pathway Analysis (IPA) of the 231 transcripts (Supplementary Table 3) showed that the top ranking functional differences regarded molecules potentially relevant for precursor cell release and trafficking from BM (Supplementary Fig. 3B,C). This upregulation involved in particular ADAM metallopeptidase with thrombospondin type 1 motif, 2 (ADAMTS2), ADAM metallopeptidase domain 20 (ADAM20) and other molecules involved in activation of matrix-metalloproteinases (MMP) or in osteoprotegerin/RANKL pathways. In addition, upregulation of transcripts coding for fibroblast growth factor 2 (FGF2), tumour necrosis factor (ligand), member 11 (TNFSF11), growth differentiation factor 6 and growth factor independent 1 were detected in PB CD34^+^DNAM-1^bright^ cells from HIV patients. On the other hand, these cells displayed decreases of transcripts involved in myeloid differentiation pathways such as B-cell CLL Lymphoma 6 (BCL6) and RAS guanyl-releasing protein 4RASGRP4.

### NK and T-cell progeny of CD34^+^DNAM-1^bright^ cells

Given the differences in transcription factor expression in Lin^−^CD34^+^DNAM-1^bright^ cells versus Lin^−^CD34^+^DNAM-1^−^UCMC, we next studied their differentiation potential using an established protocol for NK-cell *in vitro* differentiation. Cells were purified (99% purity), cultured in medium containing rhFLT3, rhSCF, rhIL-7 and rhIL-15 and analysed after 20 days of culture.

**Figure 4:**
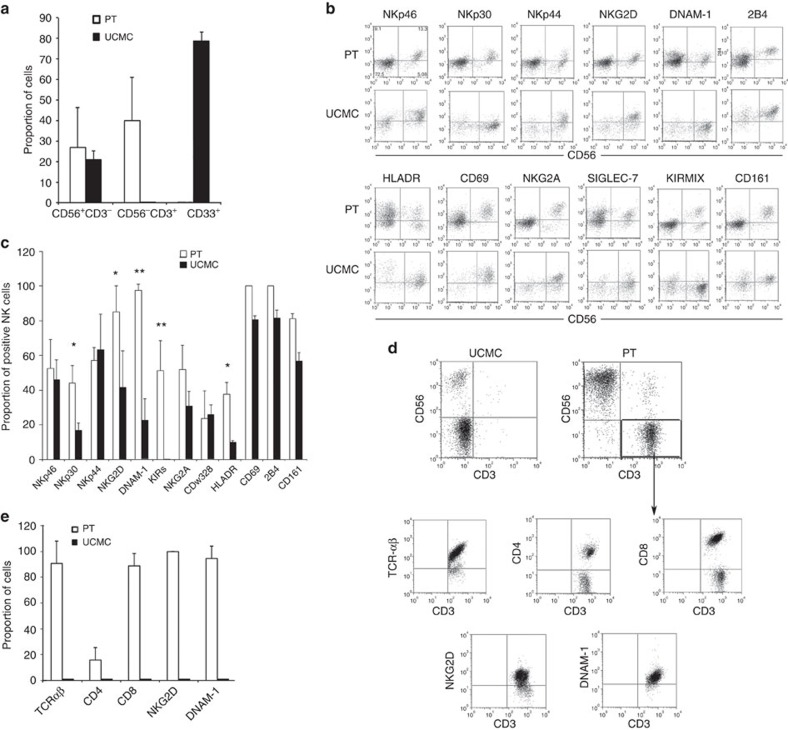
**Flow cytometric characterization of** ***in vitro*** **grown progenies of Lin**^**−**^**CD34**^**+**^**DNAM-1**^**bright**^ **PBMC.** (**a**) Analysis of T- and NK-cell proportions. Flow cytometric analysis of cells derived from sorted DNAM-1^bright^CD34^+^ PBMC from patients (PT) and from sorted DNAM-1^neg^CD34^+^ cord blood cells (UCMC). Bars express the proportion of cells recovered in culture expressing CD3, CD56 and CD33. Cells were analysed after 20 days of culture. Data are presented as mean±s.d. of 12 different experiments from different donors. CD3^+^ T cells were recovered only from DNAM-1^bright^CD34^+^-derived cultures. CD33^+^ cells of myelomonocytic lineage were derived only from sorted CD34^+^ UCMC cultures. (**b**) Flow cytometric analysis of activating and inhibitory NK-cell receptor expression on CD34-derived NK cells. Dot-plot analysis of relevant surface molecule expression in CD56^+^CD3^−^-gated NK cells derived from DNAM-1^bright^CD34^+^ patient PBMCs (PT) and from umbilical cord blood-derived CD34^+^ cells (UCMC). Representative of 20 experiments. (**c**) Analysis of receptor expression on NK cells derived *in vitro* from patient (PT) peripheral blood DNAM-1^bright^CD34^+^ cells or from healthy donor umbilical cord blood CD34^+^ cells (UCMC). Bars show the proportion of activating and inhibitory NK-cell receptor expression after 20 days of culture. Bars represent mean±s.d. expression. *P<0.05; **P<0.01 (Mann–Whitney *U*-test). (12 different experiments/patients). (**d**) Flow cytometric analysis of CD34-derived cell progeny grown *in vitro* for the expression of T-cell molecules. Dot-plot analysis of the expression of CD3, CD56, CD4, CD8, a/bTCR, NKG2D and DNAM-1 on cells derived from peripheral blood DNAM-1^bright^CD34^+^ cells (PT) or from cord blood-derived CD34^+^ cells (UCMC) is shown. CD3^+^CD56-gated cells were then studied for the expression of TCR, CD4, CD8, NKG2D and DNAM-1. 10,000 events were acquired at 20 days of culture. Dot-plots show logarithmic scale. No T cells were present in CD34^+^ UCMC-derived cultures (representative of 12 experiments). (**e**) Analysis of surface marker expression on T cells derived *in vitro* from patient peripheral blood DNAM-1^bright^CD34^+^ cells (PT) and from cord blood-derived CD34^+^ cells (UCMC). Bars show surface marker expression on CD3^+^ T cells selected after 20 days of culture (mean±s.d., 12 experiments). No T cells could be grown from CD34^+^ UCMC.

Flow cytometric analysis of cultures derived from Lin^−^CD34^+^DNAM-1^bright^ cells revealed the presence of distinct CD56^+^CD3^−^, CD56^−^CD3^+^ and CD56^+^CD3^+^ cell populations. No CD33^+^CD56^−^CD3^−^ cells of monocyte/myelomonocytic lineage could be detected. On the contrary, in cultures containing CD34^+^UCMC only, CD33^−^CD56^+^CD3^−^ (NK) and CD33^+^CD56^−^CD3^−^ (myeloid) populations were found (Fig. 4a). These data are in line with those from transcriptional and microarray analysis.

With regard to CD56^+^CD3^−^ NK cells derived from CD34^+^DNAM-1^bright^ progenitors, these consisted of a predominance of CD56^bright^ cells and in a smaller but sizeable population of CD56^dim^ cells (68.6±14.86% and 31.7±16.2%, respectively; mean±s.d.). Surface expression of NK-cell markers/receptors including NKp30, NKp44, NKp46, DNAM-1, NKG2D, p75/AIRM1 (CDw328), HLA-DR, 2B4, CD69, NKG2A and KIRs was detected. CXCR4 was not expressed (Fig. 4b). On the contrary, cell cultures derived from CD34^+^DNAM-1^−^ UCMC exclusively yielded CD56^bright^ NK cells which, in agreement with previous reports^29^,^30^, expressed low levels of NKp30, NKG2D, DNAM-1 and HLA-DR, but not KIRs (Fig. 4b,c).

**Figure 5:**
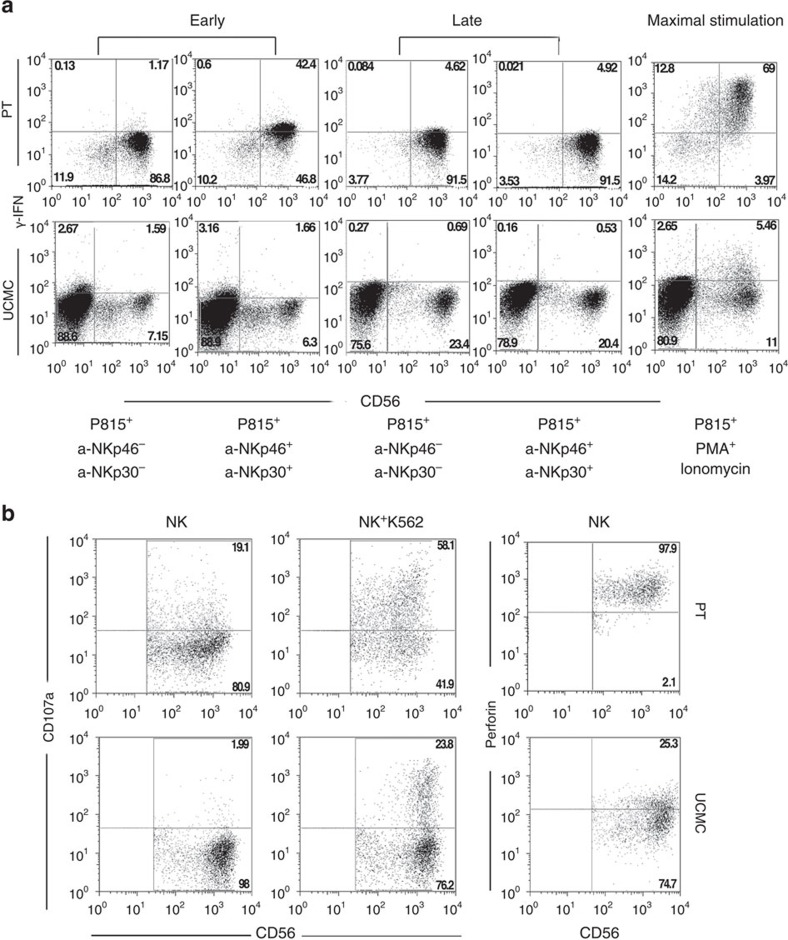
**Functional characterization of immature NK cells derived from Lin**^**−**^**CD34**^**+**^**DNAM-1**^**bright**^ **PBMC.** (**a**) IFNγ production by CD34-derived NK cells. Flow cytometric detection of IFNγ−producing NK cells growing *in vitro* from either purified patient peripheral blood DNAM-1^bright^CD34^+^ PBMC (PT) or from cord blood DNAM-1^neg^CD34^+^ cells. IFNγ production was evaluated after stimulation as occurring early (0–16 h) and late (16/20–24 h) stimulation^31^. Representative of 10 experiments. (**b**) Flow cytometric CD107a degranulation assay and perforin expression in NK cells derived *in vitro* from purified DNAM-1^bright^CD34^+^ HIV-PBMC and from purified DNAM-1^neg^CD34^+^ UCMC. NK-cell effectors were challenged with K562 target cells at 5:1 E/T ratio for 4 h. CD107a expression was detected on CD56^+^CD3^−^CD33^−^-gated cells (middle panel). Left panel: no target negative control. Right panel: intracytoplasmic perforin in NK cells derived from patient-purified CD34^+^DNAM-1^bright^ cells (PT) and from uninfected cord blood-derived CD34^+^ cells (UCMC) after 20 days of *in vitro* culture. Representative of 10 experiments.

In view of these relevant phenotypic differences in receptor expression between NK cells derived from patient PB CD34^+^DNAM^bright^CXCR4^+^ or from CB CD34^+^DNAM-1^−^CXCR4^−^, we further investigated their functional capabilities. IFN-γ production was assessed according to both early (0–16 h) and late (20–24 h) production patterns on cell triggering via natural cytotoxicity receptors (NCRs)^31^. CD34^+^DNAM-1^bright^-derived NK cells displayed early (0–16 h) IFN-γ production (Fig. 5a) and cytotoxic activity against K562 target cells (Fig. 5b). In contrast, CD34^+^ UCMC-derived iNK cells did not produce detectable amounts of IFN-γ (Fig. 5a), in line with previous reports^32^. Thus, both phenotypic and functional characteristics of NK cells derived from CD34^+^DNAM-1^bright^ cells clearly indicate a higher degree of maturation as compared with those derived from CD34^+^DNAM-1^−^CXCR4^−^ UCMC.

With regard to CD56^−^CD3^+^ cells derived from CD34^+^DNAM-1^bright^ PBMC, they expressed TCRα/β, thus confirming their T-cell identity (Fig. 4d,e). Notably, most of these T cells were characterized by an unusual phenotype as they expressed high levels of both DNAM-1 and NKG2D (Fig. 4d,e). In line with previous reports^8^,^29^,^30^, CD34^+^DNAM-1^−^ UCMC failed to give rise to T cells under the same culture conditions (Fig. 4d,e). We next excluded the possibility that detected T cells were derived from carryover of T-cells during sorting. To this end, sort purity was repeatedly verified by flow cytometry. No CD3 staining was identified after CD34^+^ cell sorting in six repeated experiments from different donors. In addition, CD3^+^ T cells were only generated in cultures of CD34^+^ cells sorted from patient PBMC but not from UCMC (Fig. 4a,d,e). More importantly, limiting dilution experiments with purified CD34^+^DNAM-1^bright^ PBMC revealed the presence of T-cell growth even at low cell numbers (10 cells per well), that is, well below the possible T-cell contamination threshold. In addition, the resulting T cells displayed a peculiar phenotype, virtually absent in PB T cells, thus further excluding PB T-cell carryover in the purified CD34^+^ cell preparations. Indeed both CD8^+^ cells (representing the majority of T cells recovered) and CD4^+^ cells, expressed NKG2D and were DNAM-1^bright^. In agreement with data from transcription factor analysis, these results show that T cells recovered after culture derive from CD34^+^DNAM-1^bright^ cells, in line with recent reports showing the existence in PB of very low levels of common T/NK-cell precursors^33^,^34^.

Taken together these results show that CD34^+^DNAM^bright^ PBMC isolated from patients with residual chronic inflammation and successfully treated HIV infection display unique phenotypic features and can generate *in vitro* both NK and T cells with peculiar, previously undescribed, characteristics.

### CD34^+^DNAM-1^bright^ correlate with T and NK cells

**Figure 6:**
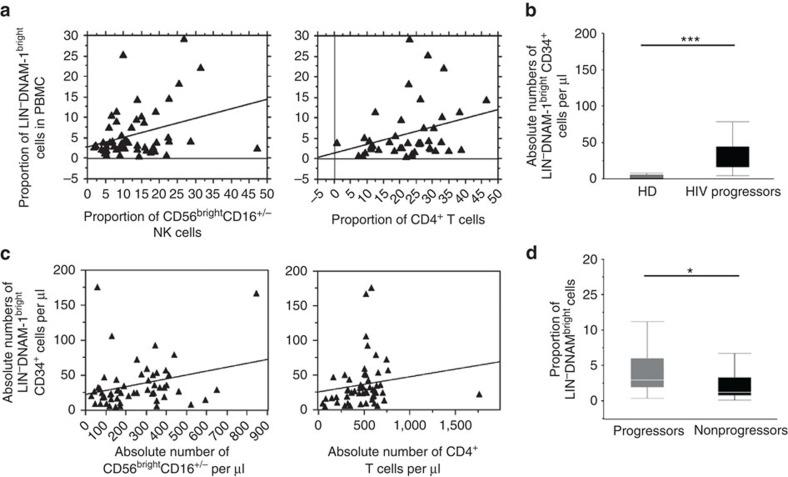
**Analysis of Lin**^**−**^**CD34**^**+**^**DNAM-1**^**bright**^ **PBMC by disease course.** (**a**) Correlation between the proportion of Lin^−^DNAM^bright^(CD34^+^) cells and CD56^bright^ NK-cell subset and CD4 T cells in HIV patients. Correlation analysis among the proportion of Lin^−^DNAM^bright^ cells, the proportion of CD4^+^ T cells and of CD56^bright^ NK cells at the same time point in aviremic cART-treated patients (n°=60). Flow cytometric data were correlated by Spearman's *ρ* test. A direct correlation was detected for both CD4^+^ T and CD56^bright^ NK cells *P<0.05. (**b**) Absolute numbers of circulating Lin^−^DNAM^bright^(CD34^+^) cells are increased in HIV-patient PBMC. Absolute numbers were derived from the proportion of Lin^−^DNAM^bright^(CD34^+^) cells and the absolute number of lymphocytes in complete blood counts on the same sample. Box-plots show increased numbers in patients with low to undetectable levels in HD (***P<0.001, Mann–Whitney *U*-test, 20 cART-treated progressors and 20 HD). (**c**) Correlation between the absolute numbers of Lin^−^DNAM^bright^(CD34^+^) cells and CD56^bright^ NK-cell subset and CD4 T cells in HIV patients. Correlation analysis among the absolute numbers of Lin^−^DNAM^bright^ cells, of CD4^+^ T cells and of CD56^bright^ NK cells at the same time point in aviremic cART-treated patients (n°=60). Flow cytometric data were correlated by Spearman's ρ test. A direct correlation was detected for both CD4^+^ T and CD56^bright^ NK cells, *P<0.05. (**d**) HIV-infected, cART-treated patients display increased circulating proportions of Lin^−^DNAM^bright^ cells compared with spontaneous HIV controller patients. The proportion of DNAM-1^bright^CD56^−^(CD34^+^) cells on CD3^−^CD14^−^CD19^−^-gated PBMCs were determined by flow cytometry in spontaneous HIV controller patients with long-term non-progressor or Elite controller disease course^35^ (non-progressors, black box) and in aviremic cART-treated patients (progressors, grey box). Box-plot analysis shows twenty-fifth and seventy-fifth percentiles (box) with median (line); vertical lines express s.d.; *P<0.05, Mann–Whitney *U*-test. (20 cART-treated progressor and 20 non-progressor patients).

In view of the relevant interindividual variability in Lin^−^DNAM-1^bright^ cell proportions in cART-treated aviremic HIV patients (0.5–40% of CD3^−^14^−^19^−^ PBMC), we further investigated whether they could associate with individual differences in NK/T-cell proportions observed after successful cART suppressing viremia below detection. Among peripheral NK cells, the CD56^bright^CD16^+/−^ cells represent the subset most proximal to CD34^+^ precursors and only later develop into CD56^dim^CD16^+^ cells. Among our patients, a direct correlation was observed between the proportion of CD34^+^DNAM-1-1^bright^ cells and those of CD56^bright^CD16^+/−^ NK cells (**P=0.0035, Spearman's *ρ*; Fig. 6a). In addition, also CD4^+^T-cell proportions directly correlated with CD34^+^DNAM-1^bright^ cell levels (*P=0.0391; Fig. 6a). To further verify that these cell expansions do not simply reflect a relative increase due to imbalances in other immune cells, we determined also their absolute numbers. Absolute numbers of CD34^+^DNAM-1^bright^ cells were significantly increased in HIV patients compared with HD (35.68±34.15 per μl versus 4.31±9.94 per μl respectively, *P*<0.001, Fig. 6b). In addition, in line with their proportions, also their absolute numbers directly correlated with both CD4^+^ T-cell numbers and with CD56^bright^CD16^+/−^ NK-cell numbers in HIV patients (Fig. 6c).

To further confirm that the proportion of Lin^−^CD34^+^DNAM-1^bright^ cells correlated with the disease course, their proportion was evaluated in a cohort of HIV controller patients (Elite Controller/ Long Term Non Progressor (EC/LTNP)^35^). Significantly lower proportions of circulating Lin^−^CD34^+^DNAM-1^bright^ cells were detected in EC/LTNP as compared with aviremic cART-treated progressor patients (*P<0.05; Fig. 6d). This observation therefore is in agreement with the concept that a lower output of Lin^−^CD34^+^DNAM-1^bright^ cells associates with the spontaneous control of HIV replication in HIV controller patients.

Since differences in these clinical disease courses were associated with different levels of ongoing inflammation, we sought to explore the relationship of Lin^−^CD34^+^DNAM-1^bright^ cells with systemic inflammation. Correlation analysis showed that in aviremic cART-treated HIV patients, plasma fibrinogen concentrations directly correlated with circulating CD34^+^DNAM-1^bright^ cell proportions (*P*=0.05, Spearman's *ρ*), while no association was observed for C-reactive protein.

Taken together these results show that, in chronically infected cART-treated HIV patients, different proportions and absolute numbers of circulating CD34^+^DNAM-1^bright^ cells reflect different disease courses and may be directly associated with systemic chronic inflammation.

### CD34^+^DNAM-1^bright^ cells circulate during inflammation

The above findings raised the relevant question of the actual role of systemic inflammation on the presence of CD34^+^DNAM-1^bright^ cells and of the more universal validity of the above observations in conditions other than HIV-1 infection. To address this question, we analysed by flow cytometry PBMC obtained from (a) donors with latent infection known to skew NK-cell peripheral repertoire (for example, CMV^36^,^37^ and EBV^38^); (b) donors with ongoing chronic infection known to affect both T- and NK-cell repertoires (for example, chronic HCV and post-primary active TB); and (c) donors with chronic inflammation and no ongoing infection (for example, COPD).

**Figure 7:**
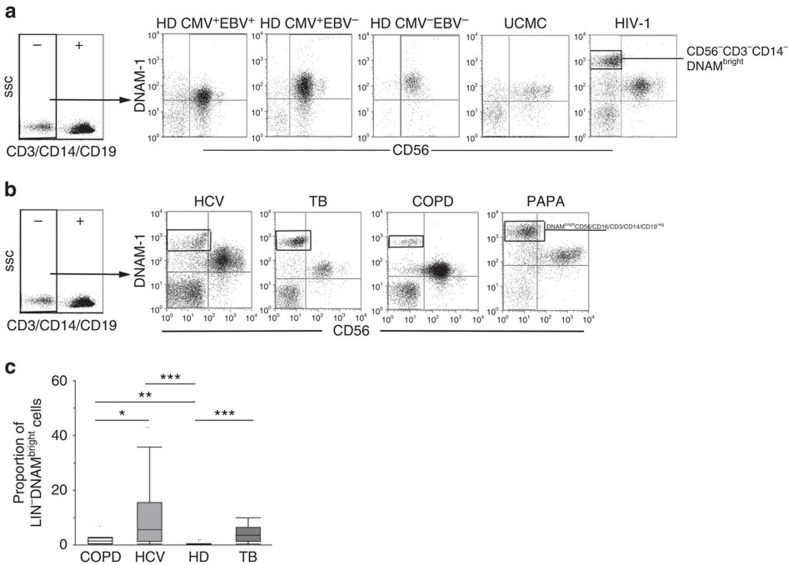
**Lin**^**−**^**CD34**^**+**^**DNAM-1**^**bright**^ **PBMC circulation occurs generally in chronic inflammatory diseases also independent of chronic infection and pathogen replication.** (**a**) Flow cytometric analysis of PBMCs from HD according to EBV and CMV serology. Selected donor PBMCs according to CMV and EBV serology were analysed. Cord blood and HIV-patient PBMC served as negative and positive control, respectively. Dot plots show DNAM-1 expression on CD3^−^CD14^−^CD19^−^CD56^+/−^-gated PBMCs. CD56^+^DNAM-1^bright^ cells are detected only in HIV patients. Representative of 20 experiments. (**b**) Flow cytometric analysis of DNAM-1 expression on fresh Lin-PBMCs from chronic HCV, acute post-primary TB, COPD patients and PAPA-syndrome patients. Lin-DNAM-1^bright^CD34^+^ cells (gates shown) were observed in HCV, TB-infected patient and in chronic obstructive pulmonary disease (COPD) CD3^−^CD14^−^CD19^−^-gated PBMCs. Representative of 67 experiments (27 HCV, 27 TB, 10 COPD and 3 PAPA syndrome PBMCs). (**c**) Different proportions of DNAM^bright^LIN^−^ cells detected in COPD, HCV, TB patients and HD donors. The proportion of DNAM^bright^Lin^−^cells was observed to increase in HCV, TB-infected patients and in COPD patients in total fresh PBMCs compared with uninfected donors. Box-plot analyses indicate twenty-fifth and seventy-fifth percentiles with median line; vertical lines express s.d.; *P<0.05 **P<0.01, ***P<0.0001, Mann–Whitney *U*-test. Representative of 64 experiments (27 HCV, 27 TB and 10 COPD).

Lin^−^DNAM-1^bright^ cells were undetectable in PB of CMV^+^ or EBV^+^ seropositive and otherwise healthy blood donors (Fig. 7a) using standard flow cytometry.

On the contrary, they were clearly present in patients with chronic active infections characterized by increased inflammation such as chronic HCV infection and post-primary TB reactivation (Fig. 7b). In these patients, the proportion of Lin^−^DNAM-1^bright^ CLP was significantly higher when compared with HD (Fig. 7c) with the highest proportions detected in viremic HCV patients. Notably, Lin^−^DNAM-1^bright^CD34^+^ PBMC were detected also in patients affected by chronic inflammatory conditions unrelated to ongoing pathogen replication such as COPD and PAPA syndrome^39^,^40^ (Fig. 7b,c). In all these conditions, Lin^−^DNAM-1^bright^CD34^+^ shared the same characteristics of those observed in HIV patients.

Thus, the presence in PB of Lin^−^CD34^+^DNAM-1^bright^CXCR4^+^-committed precursors is not associated with latent infections and is not unique to HIV-1 infection. Rather, they represent a more general condition that associates with chronic inflammation, independent of the presence/replication of pathogens.

### CD34^+^DNAM-1^bright^ cells are mobilized from BM

**Figure 8:**
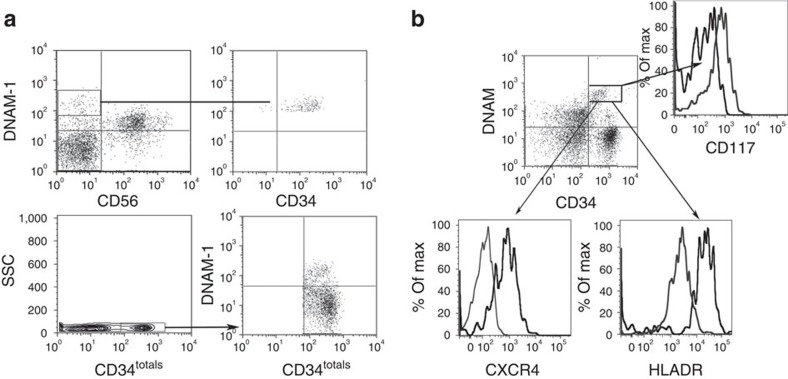
**Lin**^**−**^**CD34**^**+**^**DNAM-1**^**bright**^ **cells reside in and can be mobilized from healthy donor BM.** (**a**) Analysis of healthy bone marrow cells. Flow cytometric analysis of CD3^−^CD14^−^CD19^−^-gated BM cells shows that DNAM-1^+^CD56^−^ BM cells are CD34^+^ (upper row). Flow cytometric analysis of Lin^−^CD34^+^ cells in BM shows their expression of DNAM-1 on a subset of CD34^+^ (lower panels). Representative of 10 experiments. (**b**) DNAM-1^bright^CD34^+^ cells are present in the PBMC after HSC mobilization protocols. Flow cytometric analysis of PBMC from HSC apheresis donors (APH) following mobilization protocols (for example, G-CSF without CXCR4 antagonist drugs). DNAM-1 expression is detected on Lin^−^-gated CD34^+^ PBMC (upper panel). CD34^+^DNAM^bright^-gated APH cells express CXCR4, CD117 and HLA-DR as in CD34^+^DNAM^bright^ PBMC from HIV patients. Representative of five experiments.

CD34^+^ precursors reside in the BM and from there they seed to the secondary lymphoid organs. In our present study, the BM origin of Lin^−^DNAM-1^bright^CD34^+^ cells was suggested by their surface expression of CXCR4. Therefore, we investigated their possible presence in BM samples from HD. Flow cytometric analysis of BM cells revealed that 10% of Lin^−^CD34^+^ cells co-expressed DNAM-1 (Fig. 8a). Conversely, CXCR4 was expressed on all BM CD34^+^ cells, irrespective of DNAM-1 expression. Interestingly, CD34^+^DNAM-1^−^ cells from HD PBMC did not express CXCR4 (Fig. 8a), thus suggesting that under normal conditions Lin^−^CD34^+^DNAM-1^bright^CXCR4^+^ precursors are barely detectable in PB, and may be released from BM in case of need such as during chronic inflammation.

The fact that Lin^−^CD34^+^DNAM-1^bright^CXCR4^+^ precursors are present in BM, but barely detectable in PB of HD, while they are present in PB during chronic inflammation, prompted us to verify whether they can indeed be recovered in PB after mobilization from BM. To this purpose, we analysed CD34^+^ cells in six HSC donors undergoing CD34^+^ mobilization/harvest protocols for transplantation purposes. In these PB samples, CD34^+^DNAM-1^bright^ cells could be detected, representing 15.6±7.66% (mean±s.d.) of total CD34^+^ PB cells. Their phenotype was identical to that of Lin-CD34^+^DNAM-1^bright^CXCR4^+^ cells present in BM and in patient PBMC (HIV, HCV, TB, COPD and PAPA) (Fig. 8b).

Taken together, these findings indicate that CD34^+^CXCR4^+^DNAM-1^bright^ cells are resident in BM, and may be mobilized from there on appropriate stimuli, which may include chronic inflammation.

## Discussion

In the present study, we have characterized a previously unidentified subset of CD34^+^ cells that expresses high density DNAM-1 molecules and circulates in patients with chronic inflammatory conditions both related and unrelated to chronic infection. Remarkably, these cells represented the majority of circulating CD34^+^ cells in these patients. On the contrary, in healthy individuals they are barely detectable in the PBMC by standard flow cytometry while they reside and are readily detectable in the BM and can enter the circulation following, for example, CD34^+^ harvest protocols for HSC transplantation. Different from CB-derived CD34^+^ cells, CD34^+^DNAM-1^bright^ cells differentiate exclusively into lymphoid and not myeloid progenies, thus representing CLPs.

In human NK cells, DNAM-1 (CD226) functions primarily as an activating receptor involved in killing of different tumour targets and in NK-DC crosstalk. However, an additional functional activity of DNAM-1 is represented by its involvement in transendothelial cell migration on interaction with its ligand PVR (CD155)^41^. The finding of high levels of DNAM-1 expression on CD34^+^DNAM-1^bright^ cells supports the notion that DNAM-1 may play a role in the migration of these precursors from BM.

Recent reports would indicate that T and NK cells are generated from T/NK common progenitors^33^,^42^. Indeed, in human bipotent T/NK progenitors could be identified in PB of HDs, *albeit* at very low frequencies^34^. In agreement with these reports, and different from CB CD34^+^cells^8^,^30^, CD34^+^DNAM-1^bright^ cells could give rise to mature NK and T cells but not to myelomonocytes, and expressed transcription factors associated with T- and NK-cell maturation. Microarray analysis further confirmed that transcripts for myeloid differentiation (for example, BCL6 and RASGRP4) were shut off in CD34^+^DNAM-1^bright^ cells.

Quiescent HSCs reside in perivascular niches in which different cell types express factors that promote HSC maintenance^43^. These niches are associated with small arterioles in the endosteal BM^44^. The production of CXCL12 by cells present in the perivascular region including stromal cells, sinusoidal endothelial cells and mesenchymal progenitors, has been shown to support HSC retention. Accordingly, CXCL12 deletion in mice results in constitutive HSC mobilization^45^. HSCs have been shown to occupy perivascular niches in mice models, while early CLP would rather occupy an endosteal niche^46^. Chronic inflammation is associated with bone remodelling including endosteal niches, as a result of cytokine-induced modulation of the cells responsible for MMP-9/CXCR4-dependent HSC retention^47^,^48^,^49^. Inflammatory stimuli are thought to regulate CXCL12 expression^50^ and loss of CXCR4 leading to lymphocyte release from BM sinusoids^51^. Our present finding of circulating CXCR4^+^HLA-DR^+^CD34^+^DNAM-1^bright^ precursors in patients with chronic inflammation, suggests that they represent recent BM migrants, possibly derived from endosteal niches, following chronic inflammation with bone remodelling^47^,^48^,^49^,^52^,^53^. Thus, the ‘emergency exit' of CXCR4^+^ CLP needs to be added to established models of mature cell exit from BM.

Support for this interpretation is lent by a number of observations. First, persisting chronic inflammation is observed not only in viremic HIV patients, but also following successful cART treatment and undetectable viremia^54^, as well as HCV infection, TB, COPD and PAPA syndrome^39^,^52^,^53^. Second, the unique expression of CXCR4 in CD34^+^DNAM-1^bright^ cells, but not in CD34^+^DNAM-1^−^ cells, present in PB and in CB from healthy uninfected individuals, supports their recent and continuous release from BM. Third, microarray analysis revealed that among the 231 gene-signature-differentiating CD34^+^progenitors (CB-UCMC) from CD34^+^DNAM-1^bright^ PB precursors in patients, the activation of both MMPs (including ADAM20 and ADAMTS2) and of osteoprotegerin/RANKL pathways (for example, TNFSF11 and FGF2) represents a distinctive feature of CD34^+^DNAM-1^bright^ progenitors. This observation is in line with the MMP/CXCR4-induced CD34^+^-cell release from the BM^49^,^55^ and with the osteoclast activation induced by membrane-bound RANKL^21^,^47^. Fourth, the detection of CD34^+^DNAM-1^bright^ cells in the PB of HSC donors undergoing protocols of G-CSF-induced CD34^+^ cell mobilization/harvest provides a direct evidence that these cells are indeed mobilized from BM. Notably, in these HSC donors CD34^+^DNAM-1^bright^, cells represent only a fraction (15%) of mobilized CD34^+^ cells, while their migration appears to be more selective in patients with chronic inflammation, where they represent the majority (70-90%) of peripheral CD34^+^ cells. Finally, the finding of a direct correlation between the proportion of CD34^+^DNAM-1^bright^ cells and those of CD56^bright^CD16^+/−^NK cells and of CD4^+^ T cells in HIV patients supports the notion that these precursors may contribute to peripheral T- and NK-cell homeostasis. This homeostatic mechanism may be required when peripheral cell turnover is increased. Continuous HIV clearance by the immune system and virus generation parallels (and contributes to) the continuous CD4^+^ T-cell destruction/*de novo* production and innate and adaptive immune activation. During successful cART, virus replication does not stop completely and a spectrum of plasma virus concentrations <50 copies per ml is consistently detected. Virus is still detectable and replicates in secondary lymphoid organs including lymph nodes and gut-associated lymphoid tissue where insufficient antiviral drug levels are reached^56^. The present finding of a direct correlation between CD34^+^DNAM-1^bright^ cells and CD4^+^ T lymphocytes or CD56^bright^ NK cells could therefore reflect a continuous requirement of precursor ‘emergency exit' to support CD4^+^ T cells in the presence of persistent viral replication in lymphoid tissues. Thus, the success in maintaining CD4^+^ numbers in patients would occur at the cost of continuous, higher, more efficient ‘emergency exit' of CD34^+^DNAM-1^bright^ cells from BM niches. Accordingly, lower numbers of circulating CD34^+^DNAM-1^bright^ cells may accompany immunological non-response with insufficient CD4^+^ T-cell reconstitution on successful cART. Low CD34^+^DNAM-1^bright^ cells with low CD4^+^ T-cell numbers could also herald BM exhaustion in line with previous reports on total CD34^+^ cells^27^.

Notably, patients spontaneously controlling HIV replication and displaying conserved CD4^+^ T-cell numbers and NK function^35^ (that is, LTNP and Elite controllers) had lower proportions of CD34^+^DNAM-1^bright^ cells. Accordingly, the levels of these cells in patients with different clinical conditions may reflect differences in the intensity of chronic inflammation and in peripheral lymphocyte turnover.

While our present study was mainly focalized on the phenotypic and functional characterization of the newly identified Lin^−^CD34^+^DNAM-1^bright^CXCR4^+^ population and their relationship with chronic inflammation, several important questions remain to be answered. For example, it is unclear whether a common molecular mechanism exists for precursor mobilization in systemic inflammation and in BM mobilization for HSCT. At a first glance, this possibility seems unlikely since during chronic inflammation CD34^+^DNAM-1^bright^ cells prevail among peripheral CD34^+^ cells and may represent up to 90% of the circulating CD34^+^ cell fraction. On the contrary, during BM mobilization via G-CSF infusion, CD34^+^DNAM-1^bright^ cells represent only ∼15% of CD34^+^ cells in PBMC. Accordingly, G-CSF would not induce a selective but rather a non selective CD34^+^ cell mobilization (including both CD34^+^DNAM-1^bright^ and CD34^+^DNAM-1 cells in the same proportion present in BM). Mediators other than G-CSF may contribute to CD34^+^DNAM-1^bright^ cell mobilization from BM, although it cannot be excluded that different routes of G-CSF action (for example, systemic in the case of BM mobilization versus local in the case of BM niches in chronic inflammation) may be involved. We did not determine the G-CSF plasma levels in our patients, however, it is established that during HIV infection G-CSF concentrations are increased^57^,^58^, and this occurs also in chronic inflammatory conditions^59^,^60^. In future studies, it will be important to evaluate whether G-CSF plasma levels correlate with CD34^+^DNAM-1^bright^ cell numbers or rather whether local (that is, BM) inflammatory signals, including G-CSF, are responsible for the selective mobilization of these cells from their niches.

Next, the question is open on where Lin^−^CD34^+^DNAM-1^bright^CXCR4^+^ cells fit with the known human progenitor hierarchy^61^,^62^ and why very low levels of circulating common lymphoid progenitors (T and NK) are detectable in HDs^34^. Hierarchical development of human haemopoietic progenitors has been studied by phenotypic and molecular fingerprinting by sampling CD34^+^ cells derived from both CB and BM. Although type of sample selection may have included Lin^−^CD34^+^DNAM-1^bright^CXCR4^+^ cells in the case of BM samples, these cells may have escaped detection due to underrepresentation. According to the study by Doulatov *et al*.^61^, Lin^−^CD34^+^DNAM-1^bright^CXCR4^+^ cells would (surprisingly) fit in the group of megakaryocyte/erythroid precursors, characterized by the CD38^+^CD10-CD7-Flt3 phenotype similar to that of Lin^−^CD34^+^DNAM-1^bright^CXCR4^+^ cells. We did not analyse culture conditions favouring different pathways of these precursor differentiation. Therefore, we cannot exclude the fact that under appropriate conditions other lineage progenies may be obtained. Alternatively, in view of their low/absent expression of CD10, Lin^−^CD34^+^DNAM-1^bright^CXCR4^+^ cells could represent additional, new committed progenitors in addition to B-NK precursors^61^,^63^. With regard to circulating CD34^+^ T-/NK-cell precursors described in PB of HD by Kyoizumi *et al*.^34^, no data are available on the expression of DNAM-1/CXCR4 by these cells. For this reason, direct comparisons are not warranted. With this in mind, it is reasonable to speculate that the CD34^+^ isolated cells described by Kyoizumi *et al*.^34^ may comprise also the presently described Lin^−^CD34^+^DNAM-1^bright^CXCR4^+^. Accordingly, since Lin^−^CD34^+^DNAM-1^bright^CXCR4^+^ cells represent only a minimal fraction of HD CD34^+^ cells, it cannot be excluded that imbalances of the relative proportions of circulating CD34^+^ cell subsets (that is, CD34^+^DNAM-1-CXCR4- versus Lin^−^CD34^+^DNAM-1^bright^CXCR4^+^) reported in this work could be associated with the reported lower T-cell yield from CD34^+^ PB cells detectable with aging^34^.

Finally, it will be important to clarify whether the proportion of CD34^+^DNAM-1^bright^ cells may allow to understand whether these cells could represent a new clinical correlate reflecting differences in disease activity and course or may provide information on the response to treatment. In addition, the ability of these precursors to give rise to T lymphocytes, in addition to NK cells, in an apparently thymic-independent manner clearly requires careful further investigation.

In conclusion, the present study, in addition to identifying a previously unidentified subset of CD34^+^ precursors, sheds some light on the effect of chronic inflammation on the selective mobilization of such precursors from BM to PB. In addition, it provides means to exploit this finding for diagnostic and, possibly, prognostic purposes. Finally, it offers new important clues to better understand the pathophysiology of life-threatening diseases.

## Methods

### Patients

Samples from 60 cART-treated, virologically suppressed (HIV-RNA, viral load <50 copies per ml), HIV-infected patients enroled in multicentre observational study were evaluated after providing informed consent. IRB Approval: IRCCS AOU San Martino-IST Genova n51/09, ALS 2 n10/2011. PBMCs were also obtained from patients with PAPA syndrome (n.3, G Gaslini Institute, Genoa, Italy), HCV-infected patients (n°27) enroled in immunological monitoring of optimized treatment, acute post-primary pulmonary TB patients (n°27), COPD patients without acute exacerbation (n°10) all at the Department of Internal Medicine, the University of Genova. UCB samples (n°10) were collected at the department of Gynaecology, G Gaslini Institute from full-term newborns upon mothers' informed consent. PB of 15 healthy uninfected donors was obtained from the blood bank (G Gaslini Institute) and surplus material from 10 healthy BM samples was collected at the Hematologic and Bone Marrow Transplantation Unit, IRCCS IST San Martino Hospital (Genoa, Italy). HD apheresis samples (APH) after haematopoietic stem cell mobilization with G-CFS w/o CCR4 antagonist drugs were kindly provided by Prof. Franco Locatelli (Department of Paediatric Haematology-Oncology, IRCCS Ospedale Bambino Gesù, Rome, Italy). Immune recovery was defined as an increase in CD4^+^ T-cell counts at the time of analysis of at least 250 per μl compared with historic nadirs, or to ⩾300 CD4^+^ per μl for patients with CD4^+^ nadirs ≤100 per ml.

### Antibodies

A complete list of all the mAbs purchased is indicated in Supplementary Table 4. Anti-NKp46 (BAB281, IgG1), anti-NKp30 (7A6, IgG1), anti-NKp44 (Z231, IgG1), anti-NKG2D (BAT221, IgG1), anti-DNAM-1 (F22, IgG1), anti-KIR2DL2/S2 (CD158b1/CD158j), anti-KIR3DL1 (CD158e1), anti-KIR2DL1/S1 (CD158b/CD158 h) (mixture GL183, Z27, 11pb6, IgG1), anti-NKG2A (Z270, IgG1; Z199, IgG2a), anti-CD94 (XA185, IgG1), anti-CD85j (F278, IgG1), anti-2B4 (ST39, IgG2a), anti-CDw328 (QA79, IgG1) and anti-CD161 (MA311, IgG1) were all produced in the laboratory (A. Moretta, Genova). Anti HLA-DR (D1-12, IgG2a) was kindly provided by Dr R.S. Accolla (the University of Insubria, Varese, Italy). All mAbs were used at a final concentration of 1 μg ml^−1^.

### Cell culture

PBMCs, UCMCs, BM cells and APH cells were obtained by density gradient centrifugation (Ficoll-Hypaque) and cryopreserved at −86 °C until processed.

CD34^+^ cells were separated by immunomagnetic separation using CD34 MicroBead Kit (Miltenyi, Bergisch Gladbach, Germany). Highly purified Lin^−^DNAM-1^bright^ and Lin^−^DNAM-1^−^ populations were thus obtained using FACSAria (BD Biosciences) cell sorter according to the lack lineage and the expression of DNAM receptors. Purified Lin^−^DNAM^bright^ cells and immunomagnetic-isolated CD34^+^ cells were cultured as previously described^64^. Briefly, Lin^−^CD34^+^ cells were plated in 24-well plates (2–4 × 10^4^ per ml) in precursor complete medium with Myelocult medium (StemCell Technologies, Vancouver, British Columbia, Canada) supplemented with 10% human AB serum (ICN Pharmaceuticals Italy, Milano, Italy), 5% FCS and purified recombinant human rhIL-15, rhIL-7, SCF, FLT3-L (PeproThec, London, UK) at the final concentration of 20 ng ml^−1^. CD34^+^ cell purity was >98% as determined by flow cytometry.

### Immunofluorescence analysis

Cells were analysed by two-, three-, four- and five-colour cytofluorometry. Briefly, cells were incubated with primary mAbs, followed by phycoerythrin- or fluorescein isothiocyanate (FITC)-conjugated anti-isotype-specific goat anti-mouse secondary reagents. Direct staining was performed by fluorochrome-conjugated mAbs as a third step. For cytofluorimetric analysis, cells were gated using forward and side light scatter parameters (FACSCanto II, BD, Mountain View, CA, USA) and 10,000 events were always acquired. Mean fluorescence intensity ratios are calculated as follows: MFI sample/MFI negative control. Data were analysed using FlowJo (Tree Star, Inc.).

### CD107a degranulation assay

iNK cells were stimulated using K562-cell line at 10:1 E/T ratio in precursor complete medium. Anti-CD107a mAb (BD pharmingen) was added after 1 h. After 4 h of stimulation, cells were surface-stained using PC5-conjugated anti-CD56 and FITC-conjugated anti-CD3 (ref. 32).

### Analysis of IFN-γ and perforin intracellular production

For IFNγ production analysis, iNK cells were stimulated using FcγR^+^ mouse P815 target cells at 10:1 E/T ratio in complete medium in the absence or presence of anti-NKp30 and anti-NKp46 mAb mixture (0.1 μg ml^−1^) phorbol myristate acetate (25 ng ml^−1^, Sigma) plus ionomycin (1 μg ml^−1^, Sigma) were used for maximal IFN-γ production. GolgiPlug (BD Pharmingen) was added at 37 °C for 4 h after overnight incubation or from the start of incubation up to 16 h as previously described^31^. After incubation, iNK cells were stained with anti-CD56PeCy7 and -CD3FITC mAbs followed by permeabilization/fixation (Citofix/Citoperm protocol, BD Pharmingen) and then intracellular staining for IFN-γ in the presence of permeabilizing solution (0.1% saponin in PBS solution). About 10,000 gated events were acquired on a FACSCanto II and analysed using FlowJo software. Perforin content in iNK cells was assessed by direct stain (anti-CD56PeCy7 and -CD3FITC mAbs) followed by permeabilization/fixation as above. About 10,000 gated events were acquired on a FACSCanto II device and analysed using FlowJo software.

### May–Grunwald/Giemsa staining for cytospin slides

FACSAria-sorted Lin^−^DNAM^bright^CD34^+^ cells and PBMC cells were spinned on separated glass slides by centrifugation at 1,500 r.p.m. for 5 min. Cytospin slides were fixed with methanol and stained with May Grunwald Giemsa. Slides were then washed with distilled water and observed with an Olympus BX51 Microscope & DP70 Digital Camera System. Final optical magnification was × 400.

### RT–PCR analysis

Total RNA was extracted using RNAeasy Micro Kit (Qiagen, Hilden, Germany) from different cells: sorted CD34^+^ from HIV patients and CD34^+^ from UCMC, NK-cell populations, PHA blasts, PBMC and K562-cell line. Oligo(dT)-primed complementary DNA (cDNA) was prepared by standard technique using a Transcriptor First Strand cDNA Synthesis Kit (Roche diagnostic, Mannheim, Germany). Reverse transcription was performed at 42 °C for 10 min and at 55 °C for 50 min. PCR amplifications were carried out for 30 or 35 cycles with Platinum TAQ (Invitrogen, Carlsbad, CA) following manufacturer's instructions. Primers used were: Id2 forward 5′- AGCAAAACCCCTGTGGACG -3′, Id2 reverse 2 5′- GCTTAGATTGGGCAATTCCT -3′; E4BP4 forward 5′- CCAAGGGCCCCATCCATTC -3′, E4BP4 reverse 3 5′- ACTTTGTAGCCACTGTCTTTC -3′; T-bet forward 5′- AATGTGACCCAGATGATTGTG -3′, T-bet reverse 5′- GCATAGGGCGGAACCAGC -3′; FOXP3 forward 5′- CCCACTTACAGGCACTCCTC -3′, FOXP3 reverse 5′- CTTCTCCTTCTCCAGCACCA -3′; GATA-1 forward 5′- AGATGAATGGGCAGAACAGG -3′, GATA-1 reverse 5′- AGTGGCCGGTTCACCTGG -3′; β-actin forward 5′- ACTCCATCATGAAGTGTGACG -3′, β-actin reverse 5′- CATACTCCTGCTTGCTGATCC -3′. Annealing temperatures were 55 °C (E4BP4), 56 °C (FOXP3 and GATA-1), 58 °C (Id2 and β-actin) or 60 °C (t-bet). PCR products were separated by electrophoresis on a 1.5% agarose gel and visualized by ethidium bromide staining.

### Gene expression array

Total RNA from sorted CD34^+^ of two healthy individuals and two HIV-infected patients were extracted using miRNeasy minikit (Qiagen, Valencia, CA) according to the manufacturer's protocol. RNA quality and quantity were estimated using Nanodrop (Thermo Scientific, Pittsburgh, PA)) and Agilent 2100 Bioanalyzer (Agilent Technologies, Palo Alto, CA). First- and second-strand cDNA were synthesized from 30 ng of total RNA according to manufacturer's instructions (Nugen Ovation Pico WTA System V2, San Carlos, CA). cDNAs were fragmented, biotinylated (Nugen Encore Biotin Module, San Carlos, CA) and hybridized to the GeneChip Human Gene 1.0 ST Arrays by using WT Terminal Labelling Kit (Affymetrix WT Terminal Labelling Kit, Affymetrix, Santa Clara, CA). The arrays were washed and stained on a GeneChip Fluidics Station 450 (Affymetrix) and scanned by GeneChip Scanner 3000 (Affymetrix).

The global gene expression profiling was analysed using Partek Genomics Suite (St Louis, MO). Functional analysis was performed using the IPA (Qiagen). Microarray data have been deposited to the Gene Expression Omnibus (GEO) under the accession number GSE69557.

### Statistical analysis

Statistical analysis was performed using the Mann-Whitney U tests for unpaired datasets for comparisons. Spearman test was used for correlation analysis. Student's *t*-test was used for molecular analysis of paired datasets. Tests were two-sided. Analysis was performed using JMP 10.0 (SAS) if not otherwise stated in the text.

## Additional information

**Accession codes:** Microarray data have been deposited to the Gene Expression Omnibus (GEO) under the accession number GSE69557.

**How to cite this article**: Bozzano, F. *et al*. ‘Emergency exit' of bone-marrow-resident CD34^+^DNAM-1brightCXCR4^+^ committed lymphoid precursors during chronic infection and inflammation. *Nat. Commun.* 6:8109 doi: 10.1038/ncomms9109 (2015).

## Supplementary Material

Supplementary Information Supplementary Figures 1-3, Supplementary Tables 1-3 and Supplementary Experimental Procedures.

## Figures and Tables

**Table 1:** Comprison of surface marker expression on CD34^+^ cells in peripheral and umbilical cord blood.

**Receptors**	**CD34**^**+**^ **UCMC**	**CD34**^**+**^**DNAM**^**NEG**^ **PT**	**CD34**^**+**^**DNAM**^**BRIGHT**^ **PT**
DNAM	−	−	++
CXCR4	−	−	++
CD69	−	±	+
CD94	−	−	−
CD117	±	±	−
CD38	+	+	+
CDw328	−	−	−
CD161	−	−	−
HLA-DR	+	+	++
CD8	±	±	±
CD4	−	−	−
CD11c	−	−	−
CD123	−	−	−

UCMC, umbilical cord blood mononuclear cells.
