# Supplementary material for: ‘Emergency exit' of bone-marrow-resident CD34+DNAM-1brightCXCR4+-committed lymphoid precursors during chronic infection and inflammation
Source: Nat Commun. 2015 Oct 5;6:8109. doi: 10.1038/ncomms9109 (PMC4600731; doi:10.1038/ncomms9109)
Supplement: Supplementary Information — Supplementary Figures 1-3, Supplementary Tables 1-3 and Supplementary Experimental Procedures. [file ncomms9109-s1.pdf]

FIGURE S1

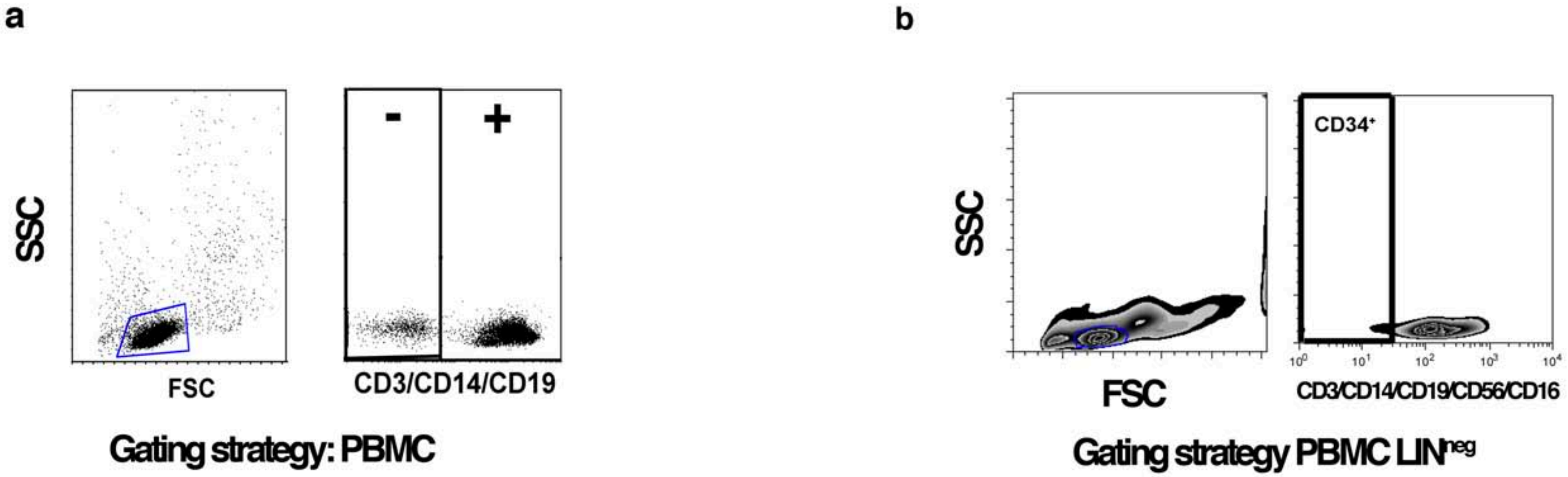

FIGURE S1. PANEL A. Gating strategy from total fresh PBMC. PBMCs FSC/SSC gated cells (left panel). DNAM-1 expression was analysed on CD3/CD14/CD19 negative-gated PBMCs (right panel). **PANEL B. Gating strategy from total fresh PBMC.** The Left dot-plot shows FSC/SSC gating strategy. To analyse DNAM-1 expression, lineage-negative gated PBMC were selected (right panel).

FIGURE S2

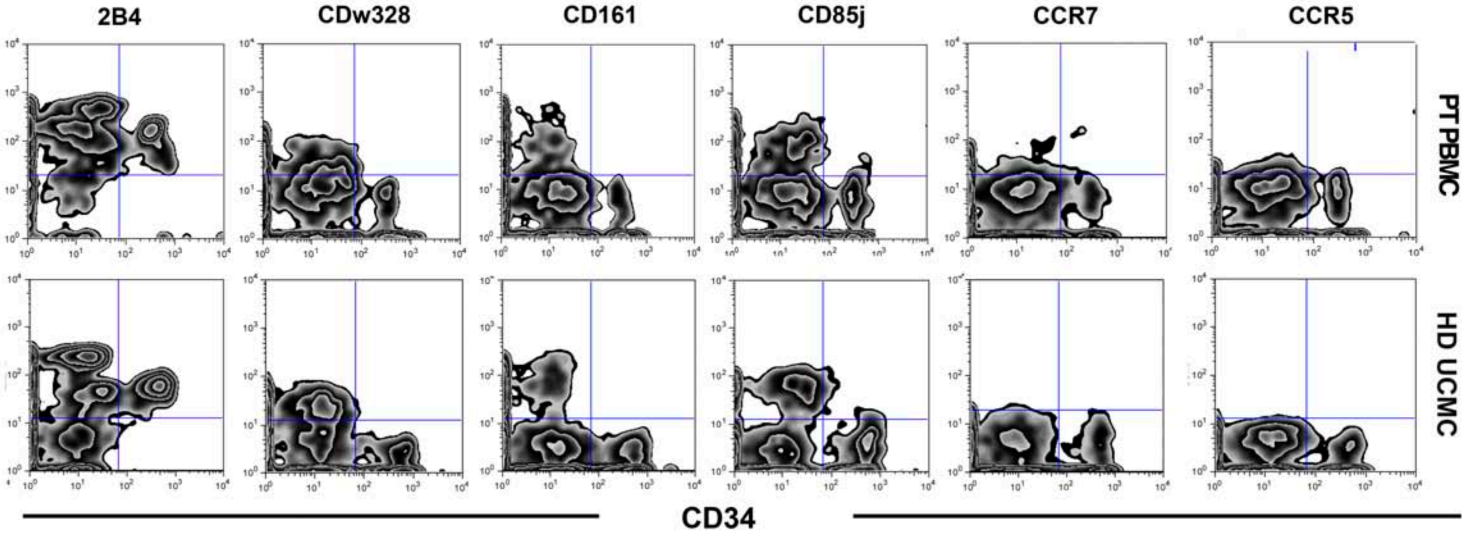

FIGURE S2. Flow cytometric analysis of Lin-DNAM-1brightCD34+ receptor expression in HIV patients (upper row), and UCMC samples (lower row). Plots show receptor expression on fresh Lineage negative gated DNAM-1brightCD34+ cells. Representative of 20 experiments.

FIGURE S3

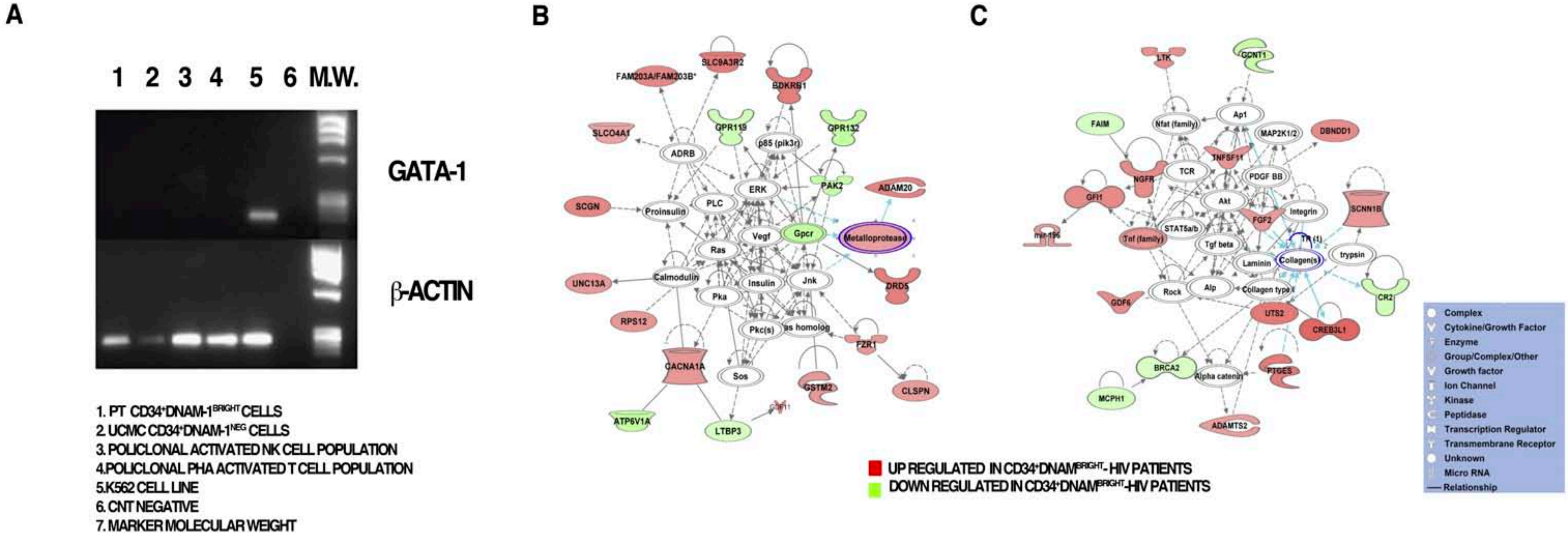

FIGURE S3 PANEL A. RT-PCR evaluation of GATA-expression on PBMC Lin-DNAM-1brightCD34+ and UCMC Lin-DNAMnegCD34+ cells. PANEL B. IPA analysis based on the 231 differentially expressed genes. Top ranking network centered on molecules involved in connective tissue development and function. **PANEL C. IPA analysis based on the 231 differentially expressed genes.** Fourth ranking network centered on molecules involved in skeletal and muscular system development and function.

Table S1. Student's t test CD34$^+$DNAM-1^{bright} -HIV patients vs. CD34$^+$DNAM-1^{bright} –UCMC samples (p<0.01). List of 150 transcripts up regulated in CD34$^+$DNAM-1^{bright} -HIV patients. Transcripts are ordered based on descending *p* value. FC represents the ratio between the gene of interest and reference gene intensity.

GENE	P-VALUE	FC%	GENE	P-VALUE	FC%
Tex13a	7.98E-08	129.388	*Hnrnpcl1*	0.00469373	148.536
Mir492	4,38E-08	124.522	*Mzt2a*	0,00471349	156.578
Mir196a2	4.48E-05	113.533	*Gdf6*	0.00475928	130.936
Cyp26a1	0.000132224	132.904	*C5orf53*	0.00477333	141.361
Ncrna00287	0.000206923	143.997	*Kif12*	0.00478594	138.007
Or5c1	0.000261569	156.763	*Znf37bp*	0.00500981	133.172
Id2b	0.000494436	118.266	*Cacna1a*	0.00504828	118.649
Gsx2	0.000571974	135.67	*Comp*	0.00507168	132.942
Alg12	0.000615813	160.573	*C8orf30A*	0.00523112	137.557
Slc41a1	0.000638324	175.728	*Rnu1-1*	0.00527147	246.526
B3gnt1	0.00066466	125.271	*Snord115-8*	0.00533547	105.765
C8orf30A	0.000707341	134.697	*Loc146429*	0.00534279	117.288
F3	0.000771731	123.425	*Krtap5-3*	0.00535338	181.831
Ampd3	0.000946379	123.237	*Tmem155*	0.00535388	133.603
Or4c15	0.00105492	127.48	*C17orf103*	0.00548564	149.718
Gfi1	0.00108954	134.081	*Tnfsf11*	0.00551009	122.051
Flj38576	0.00114032	141.439	*Znf512b*	0.00568204	147.865
Sema4f	0.00116476	116.466	*Bdkrb1*	0.00572429	149.469
Cpne4	0.00120486	156.818	*Fmnl3*	0.00574151	199.923
Ngfr	0.00124527	132.429	*Scgn*	0.00576429	139.532
Gprin2	0.00126082	158.36	*Clspn*	0.00584883	111.783
C1orf65	0.00128558	147.977	*Mgc10814*	0.00592493	164.435
Tmem158	0.00131625	111.338	*Kcnab3*	0.0059811	132.175
Kaz	0.00136806	141.184	*C20orf132*	0.00608508	136.731
Sgca	0.00137187	103.524	*Drd5*	0.00629882	148.402
Ppp4r4	0.00137671	117.532	*Unc80*	0.00632609	142.713
Kiaa1217	0.00144082	137.511	*Fzr1*	0.00642162	117.778
Dapl1	0.00152146	123.7	*Pltp*	0.00655716	138.613
Cecr6	0.00155234	122.072	*Hox83*	0.00681517	139.55
Adam12	0.00155486	141.927	*Bsx*	0.00683173	135.998
Dctpp1	0.00165736	175.161	*Gsdmd*	0.00694122	216.681
Wash2p	0.00172695	104.937	*C20orf79*	0.00696374	159.948
Jmjd4	0.00174616	148.19	*Cdc42bpg*	0.00696464	170.723
Scnn1b	0.00177777	116.608	*Dyx1c1*	0.00697627	130.471
Kprp	0.00178401	157.942	*Cenph*	0.0070141	123.599
Or9a2	0.00193228	128.269	*Sec14l5*	0.00732991	150.902
Pga5	0.00199512	129.072	*Igkc*	0.00741221	498.739
Kif26b	0.0020697	115.302	*Dnajc27*	0.00743761	122.351
Taar3	0.00212751	141.757	*Unc13a*	0,00750369	115.713
Gdf11	0.0027237	141.854	*Tmem125*	0.00754186	134.81
Loc440518	0.0023215	172.739	*Nrn1*	0.00757256	130.893
Rbp4	0.00234254	122.995	*C1qtnf9b*	0.0076313	125.341
Rasd1	0.00239113	143.518	*Spem1*	0.00770529	162.964
Susd2	0.00241844	124.346	*Dbndd1*	0.00791303	132.856
Hnf1a	0.00242176	163.62	*Flj30901*	0.00793571	158.108
Mir339	0.00258067	150.747	*Trim49*	0.00793777	132.481
Loc554202	0.00269432	129.063	*Mpped1*	0.00818464	160.267
Tubb3	0.00294842	133.189	*Barhl1*	0.00819594	122.394
Ormdl1	0.00311558	163.309	*Adam20*	0.00831109	142.944
Rps12	0.00315155	118.079	*Uts2*	0.0085748	154.667
Ctag2	0.00321666	152.107	*Hdgfrp2*	0.0085916	122.677
Gstm2	0.00328003	116.583	*Ppapdc2*	0.00862071	118.058
Tmed10p1	0.00330336	136.307	*Elovl4*	0.00868378	109.703
Susd5	0.00335294	156.272	*Flj44881*	0.00875628	135.081
C1orf64	0.00342439	128.101	*Yif1a*	0.00888212	196.563
Slc7a14	0.00352797	109.783	*Loc100128501*	0.00890827	120.312
Alox5ap	0.00358634	205.32	*C20orf200*	0.0090142	150.175
Znf502	0.00367755	105.781	*Mxra7*	0.00925757	202.441
Srrm3	0.00373844	135.426	*Mrap2*	0.00929912	168.86
Ddx43	0.00389914	157.084	*Creb3l1*	0.00933962	178.9
Trex2	0.00392557	122.692	*Cox2*	0.00938422	131.135
Znf43	0.00400056	145.454	*Wdr88*	0.00942399	139.254
Ca9	0.00409969	148.908	*Slco4a1*	0.00952966	104.897
Slc9a3r2	0.00416978	152.766	*C6orf15*	0.00955894	171.406
Svop	0.00417197	138.135	*Vwa1*	0.00956367	119.584
Loc652276	0.00419455	214.582	*Sephs2*	0.00956845	128.963
Igfbp5	0.00436105	118.031	*Asb10*	0.0095738	117.299
F12	0.00439499	136.05	*Camk2g*	0.00964728	143.84
Rnasel	0.0045117	160.497	*Eif3f*	0.00973837	151.698
Tmem132c	0.00455201	163.572	*Hmx3*	0.009747	142.94
Nub1	0.00456236	135.92	*Fgf2*	0.00980584	115.407
Ltk	0.00458008	125.142	*Nags*	0.00981052	153.619
Ticam1	0.0046207	124.35	*Cyp4f12*	0.00983179	135.707
Ptges	0.00468789	153.051	*Adamts2*	0.0099475	109.956

FC%: FOLD CHANGE PERCENTAGE

Table S2. Student's t test CD34$^+$DNAM-1^{bright}-HIV patients vs. CD34$^+$DNAM-1^{bright}-UCMC samples (*p*<0.01). List of the 81 down regulated transcripts in CD34$^+$DNAM-1^{bright} -HIV patients. Transcripts are ordered based on descending *p* value. FC is ratio between gene of interest and gene of reference intensity.

GENE	P-VALUE	FC%
Rrn3p1	0.0000142	-159.877
Ncstn	0.000389329	-241.417
Uba5	0.000707657	-222.377
Rasgrp4	0.00102627	-269.527
Dirc2	0.00114709	-352.366
Rrn3	0.00120193	-488.111
Ddx12	0.00129533	-174.884
Ddx11	0.00149541	-120.567
Mrps12	0.00149748	-226.268
Plekha9	0.0015188	-167.387
Bcl6	0.00157304	-230.209
Cr2	0.00180571	-141.669
Pms2l2	0.00196946	-133.771
Snord47	0.00209355	-266.274
Pign	0.00230905	-127.679
Pck2	0.0026385	-147.182
Spg11	0.00264728	-184.399
C14orf147	0.00272361	-165.194
Jup	0.00292185	-239.206
Utp23	0.00304437	-126.239
Gpr119	0.00310717	-146.431
Wbp2	0.00311113	-240.377
Nsd1	0.0031518	-138.5
Znf292	0.00316549	-174.639
Gosr1	0.00328058	-164.487
Gclc	0.00329156	-128.462
Tmem194b	0.00360231	-120.626
Anpep	0.00379825	-252.782
Pak2	0.00386125	-185.239
Snord56b	0.00389616	-669.974
Upk1b	0.0042882	-113.509
Scarna14	0.00430975	-104.949
Eif3h	0.00435533	-130.535
Rbm33	0.00445999	-151.322
Sgms2	0.00448476	-144.237
Scd	0.00504386	-385.918
Znf22	0.00537929	-125.733
C17orf91	0.00540071	-174.652
Atp11a	0.00543896	-310.932
Atp6v1a	0.00550731	-191.966
Crp	0.00578875	-108.221
Sptbn1	0.00584383	-284.918
Hdac5	0.00601457	-138.932
Itfg1	0.00645185	-189.887
Gcnt1	0.00666027	-174.617
Cbwd5	0.00671942	-232.412
Lpcat2	0.00678639	-253.206
C16orf68	0.00682296	-139.25
Tmem149	0.00689199	-118.601
Brca2	0.00693994	-121.961
Spdyc	0.00701194	-11.135
Mx2	0.00703677	-245.221
LTbp3	0.00744335	-125.789
Rnu6atac	0.00744681	-111.244
Snord4a	0.00748362	-216.083
Dcun1d1	0.00750533	-127.846
Lrrk2	0.00768565	-349.651
Exoc4	0.00773002	-212.821
Gnl3l	0.00784693	-161
Pibf1	0.00787627	-158.926
Wdr52	0.00788432	-150.977
Chmp2a	0.00801647	-189.297
Gpr132	0.00807259	-192.556
Tubd1	0.00812087	-110.815
Scml4	0.00814357	-172.515
Mtfr1	0.00828356	-14.068
Mcph1	0.00832308	-130.722
Ift7a	0.00851406	-121.588
Znf577	0.00857855	-151.535
Lyl1	0.00865964	-118.449
Faim	0.00866997	-114.673
Or52e2	0.00872886	-164.884
Mad2l1bp	0.00880787	-226.221
Magt1	0.00881395	-192.966
C10orf125	0.00885464	-138.024
Ptplad1	0.00888264	-157.948
Nup93	0.00939546	-203.308
C12orf40	0.00988782	-122.844
Gif	0.00990045	-114.233
Anp32b	0.00990193	-152.01
C14orf2	0.00995846	-125.964

FC%: FOLD CHANGE PERCENTAGE

Table S3. Summary of the IPA functional analyisis performed on the 231 trascripts. The top networks and top biofunctions in which the trascripts are involved are here reported.

**A Top Bio function associated with the 231 differentially expressed gene**		
Name	*p* value	# of Molecules
Skeletal and Muscular System Development and Function	8.86E-05-1.88E-02	19
Embryonic Development	2.64E-04-1.88E-02	26
Organismal Development	2.64E-04-1.88E-02	38
Tissues Development	2.64E-04-1.88E-02	34
Hematological Development and Function	5.25E-04-1.88E-02	22

SUPPLEMENTARY EXPERIMENTAL PROCEDURES.

FLUOROCHROME-CONJUGATED MABS

mAbs anti- human CD56 PeCy7: Immunotech-Coulter, Marseille, France CLONE N901 **Item N°** A21692
mAbs anti- human CD56 PeCy5: Immunotech-Coulter, Marseille, France CLONE N901 **Item N°** A07789
mAbs anti- human CD34 PeCy7: BD Pharmigen, San Jose, CA, USA CLONE 581 **CAT** 560710
mAbs anti- human CD34 FITC BD Pharmigen, San Jose, CA, USA CLONE 581 **CAT 555821**
mAbs anti- human CD38 PerCp/Cy5.5: BioLegend, San Diego, CA, USA CLONE HIT2 **CAT** 303522
mAbs anti- human CD117 PerCp/Cy5.5: BD Pharmigen, San Jose, CA, USA CLONE YB5.B8 **CAT** 562094
mAbs anti- human CD3 APC: BioLegend, San Diego, CA, USA CLONE HIT3A **CAT 300312**
mAbs anti- human CD3 FITC BioLegend, San Diego, CA, USA CLONE HIT3A **CAT 300306**
mAbs anti- human CD14 APC: BioLegend, San Diego, CA, USA CLONE M5E2 **CAT 301808**
mAbs anti- human CD19 APC: BioLegend, San Diego, CA, USA CLONE HIB19 **CAT 302212**
mAbs anti- human Integrin Beta7 APC: BD Pharmigen, San Jose, CA, USA CLONE FIB504 **CAT 551082**
mAbs anti- human CD10 APC: BD Pharmigen, San Jose, CA, USA CLONE HI10a **CAT 332777**
mAbs anti- human CCR7 APC: R&D Systems, Minneapolis, MN CLONE 150503 **CAT FAB197A-100**
mAbs anti- human CD33 FITC: BioLegend, San Diego, CA, USA CLONE HIM3-4 **CAT 303304**
mAbs anti- human CD7 FITC: BD Pharmigen, San Jose, CA, USA CLONE M-T701 **CAT 555360**
mAbs anti- human Lineage Cocktail 2 (lin2) (CD3, CD14, CD19, CD20, CD56): : BD Pharmigen, San Jose, CA, USA CLONE NCAM16.2 **CAT 643397**
mAbs anti- human CD107a PE: BD Pharmigen, San Jose, CA, USA CLONE H4A3 **CAT 555801**
mAbs anti- human CD1a PE: Beckman-Coulter, Marseille, France CLONE BL6, **CAT 082A07742**
mAbs anti- human CX3CR1 PE: MBL International, MA CLONE 2A9-1, **CAT D070-5**
mAbs anti- human CD69 PE: BioLegend, San Diego, CA, USA CLONE FN50 **CAT 310906**
mAbs anti- human CD49d (alpha 4) VIOBLUE: Miltenyi, Bergisch Gladbach, Germany CLONE MZ18-24A9 **CAT 130-099-680**
mAbs anti- human CD16 : BD Pharmigen, San Jose, CA, USA CLONE 3G8 **CAT 557758**
mAbs anti- human CD4 Purified: BD Pharmigen, San Jose, CA, USA CLONE SK3 **CAT 346320**
mAbs anti- human CD8 Purified: BD Pharmigen, San Jose, CA, USA CLONE SK1 **CAT 346310**
mAbs anti- human TCRab Purified: BD Pharmigen, San Jose, CA, USA CLONE WT31 **CAT 347770**
mAbs anti- human CXCR4 Purified: BD Pharmigen, San Jose, CA, USA CLONE 12G5 **CAT 555972**
mAbs anti- human CCR5 Purified: BD Pharmigen, San Jose, CA, USA CLONE 2D7/CCR5 **CAT 555991**
mAbs anti- human CD62L Purified: BD Pharmigen, San Jose, CA, USA CLONE DREG-56, **CAT**. 555541
mAbs anti- human CXCR1 Purified: Santa Cruz Biotechnology, Inc. USA **CAT**. sc-7303
mAbs anti- human CXCR3 Purified: R&D System, Minneapolis CLONE 49801, **CAT** MAB160
mAbs anti- human Perforin PE BioLegend, San Diego, CA, USA CLONE dG9, **CAT** 308106

ANTI-ISOTYPE-SPECIFIC GOAT ANTI-MOUSE SECONDARY REAGENTS
Goat Anti-Mouse IgG1 PE conjugated. Beckman Coulter CA, **CAT 731840**
Goat Anti-Mouse IgG2b FITC conjugated. Southern Biotech, Birmingham, AL, USA **CAT 1090-02**
